# Development of Serious Games (Equit’Game) to Address Health and Environmental Inequalities: Protocol for an App-Delivered Program to Perform a Territorial Diagnosis

**DOI:** 10.2196/11786

**Published:** 2020-01-07

**Authors:** Wahida Kihal Talantikite, Damien Renou, Arnold Magdelaine, William Harang, Denis Zmirou-Navier, Severine Deguen

**Affiliations:** 1 Laboratoire Image Ville Environnement UMR 7362 du CNRS University of Strasbourg Strasbourg France; 2 Ludiscape Vaulx-en-Velin France; 3 School of Public Health Rennes France; 4 Medical Department Lorraine University Nancy France; 5 Research Institute in Environmental and Occupational Health INSERM U1085 Rennes France; 6 Sorbonne Universités, UPMC Univ Paris 06, INSERM Institut Pierre Louis d’Epidémiologie et de Santé Publique (UMRS 1136) Department of Social Epidemiology Paris France

**Keywords:** serious game, territorial diagnosis tool, health inequality, environmental inequalities, Equit'Game

## Abstract

**Background:**

Territorial diagnosis is a prerequisite for local actions concerning public health and for the reduction of social, environmental, and health-related inequalities. To orient local programs or initiatives targeting health inequalities, policymakers need a simulation of territorial diagnosis tools. Yet, very few platforms have been developed for the purpose of guiding public authorities as they seek to reduce these social inequalities.

**Objective:**

This study aimed to describe the design and methods of the development process of a territorial diagnosis tool based on a serious game named Equit’Game that puts learners at the heart of the territorial diagnosis process, asking them to review the current state of health, environmental state, and socioeconomic state of their territory.

**Methods:**

The realistic situations employed in our serious game should encourage players, in a fun and playful manner, to (1) appropriate the data of their own territory, (2) apply their methodological knowledge in a practical way, (3) reflect on the most pertinent statistical or spatial tools for their situation, and (4) ultimately, to acquire new knowledge and skills in the use of territorial diagnosis tools with a spatial dynamic. Equit’Game was deployed over the course of a week’s training and structured into 4 levels: level 1, Dataminer (identifying relevant information to respond to the question); level 2, Analyst (selecting the appropriate method of analysis); level 3, Atlas (mapping the data); and level 4, Cluster (extraction of statistical and spatial information). Equit’Game has also been designed as a sort of virtual campus, creating a fun learning environment in which each door represents a level. Users can access Equit’Game via a platform compatible with tablets, PCs, and mobile phones.

**Results:**

In the first step, we tested our application interface designed especially for adults among a panel of local health professionals. The following are some of the most relevant points: font size and colors used, voice accompaniment in texts and messages guiding the user, clear and easy interfaces, and the change between successive game levels. In the second step, we used our application, Equit’Game, with postgraduate students from the School of Public Health (École des hautes études en santé publique). At the end of the game session, we conducted a satisfaction survey, including several items covering both the application interface and the execution of the game.

**Conclusions:**

Equit’Game was developed to help learners with the techniques of territorial diagnosis, with the aim of creating an innovative tool for public health capable of conveying educational messages and providing a structure for training.

**International Registered Report Identifier (IRRID):**

PRR1-10.2196/11786

## Introduction

Public policy on health and social matters insists that territorial diagnosis be a prerequisite for all territorial procedures and, on a more general level, for all local development actions [1]. This process involves stakeholders with interests and decision-making powers, which are different but complementary; coordinating their actions is one of the key factors determining the success or the failure of local projects.

Furthermore—to more effectively steer local actions concerning public health and the reduction of social, environmental, and health-related inequalities—it is crucial that we fully understand the socioterritorial constructions that serve to perpetuate or even aggravate health-related inequality.

A review of the extant research conducted with the direct or indirect involvement of local authorities (metropolitan areas or regions) leads us to the following observation: faced with socioterritorial health inequality, territorial stakeholders (elected official and institutions) run up against the same obstacles, particularly the absence of tools for more effectively scheduling and targeting actions on the ground.

For instance, in France, in its evaluation of France’s second National Health and Environment Plan (PNSE2), the High Council of Public Health (Haut Conseil de santé publique) clearly highlighted the lack of sufficient tools to help territorial authorities to prioritize and to target their actions on the ground, and thus effectively reduce social and environmental inequality [2].

Researchers also have a role to play in this field, because the absence of tools and methodologies enabling concrete and efficient responses to these local priorities is a significant problem. Moreover, an increasing number of research projects have documented the existence of social inequalities in health [3,4] and, to a lesser extent, the existence of environmental inequalities [5-7]. Yet, very few platforms have been developed for guiding public authorities as they seek to reduce these social inequalities. It appears to us that establishing a full and precise social and health-related diagnosis at the local level is a crucial requirement if we want to optimize and prioritize the use of resources, directing them toward those categories of citizens who accumulate multiple risk factors.

As such, the dissemination of research findings and the transfer of knowledge to establish training and educational targets and to simulate situations that closely reflect reality appears to be a pertinent and promising strategy for spreading the techniques of territorial diagnosis.

It was in this spirit that the *Equit’Game* tool was created; it forms a part of the Equit’Area project [8], focusing on the issue of social inequalities in health.

The primary objectives of Equit’Game are as follows: on the one hand, to teach the methods and approaches required to perform territorial analysis and, on the other hand, to put learners in life-like situations. The idea is to put learners at the heart of the territorial diagnosis process, asking them to review the current state of health, environmental state, and socioeconomic state of their territory while completing a number of compulsory *levels*; these levels ensure that all of the key steps in the process of territorial diagnosis are covered.

This professionally oriented teaching will provide a theoretical framework of realistic situations derived from the Equit’Area research program [9,10], preparing learners to contribute to the creation of similar projects in their own territories.

This paper is divided into several sections: Section 1, Introduction reviews the existing literature on the use of serious games to promote health; Section 3 provides more details on the methods used, the participants, and the design of the game; and Section 4 details the technical results. Finally, Section 5 contains our conclusions and perspectives for future additional development.

The literature review dealing with serious games reveals a multitude of definitions and classifications, which reflect the diversity of approaches and perspectives found in the different sectors concerned (education, media, health, and simulators). Nevertheless, the most widely shared definition seems to be the one written by game designers, Sande Chen and David Michael: “These games have an explicit and carefully thought-out educational purpose, and are not intended to be played primarily for amusement.”[11]

In the definition provided by Lelardeu et al [12], the various objectives of these serious games can be divided into 3 main categories: (1) spreading a message, (2) providing training (eg, exergames), and 3) promoting data exchange (eg, datagames).

Among serious games designed to spread a message, we can identify several types of messages: teaching and education (eg, edugames), sharing information (eg, newsgames), and also more marketing-persuasive messages intended to influence or induce certain behavior (eg, advergames).

Since the concept of serious games was first developed, this form of learning has been deployed for users from different sectors, from military personnel to teachers and health care professionals [13]. In the health care sector, since 2002, several serious games have been created, focusing on physical activity, rehabilitation, cognitive stimulation, surgery, or emergency care for both patients and professionals [14-16].

Most serious games developed in recent years are designed to help teach effective decision making in health care matters, making them *an innovative tool for public health*.

As such, modeling of health information may play a role in the creation of tools to be used both for training and decision making, for example, a serious game that can be used to make diagnoses.

This harks back to the definition offered by Abt [17] over 40 years ago in his work, *Serious Games*: “A game is an activity among two or more independent decision-makers seeking to achieve their objectives in some limiting context.”

The tools we are dealing with place learners within a given defined context, obliging them to make choices and decisions [18]; the aim is to push users to adapt their responses to certain situations and then conduct them to transfer these lessons to their day-to-day professional activities.

To make the game environment as realistic as possible, some serious games seek to incorporate data and results based on scientific research, presented in a format that is sufficiently accessible and comprehensible to be used in the decision-making process. One illustration of this approach is the *Time After* application, developed by Reichlin et al in 2011 [19].

An increasing number of public institutions are taking the decisive step to commission serious games, for example, the World Health Organization’s yellow fever epidemic application aimed at health care professionals. This app lets players manage various resources and perform actions to control yellow fever epidemics. The review of the existing literature conducted by Ohannessian et al [20] clearly explains that serious games focusing on vaccinations could be a useful educational tool for decision makers.

In France, serious games are beginning to gain traction with territorial authorities. One recent example is the serious game named *Ecoville* developed by the Agency for the Environment and Energy Management (Agence de l’Environnement et de la Maîtrise de l’Énergie [ADEME]), which invites users to create their own environmentally friendly town and to reflect on the consequences of urban sprawl and mixed habitats while learning about the importance of energy management and respecting the environment [21]. A recent addition, again from ADEME, is the application, *Réflexe planétaire*, which aims to educate users in an entertaining way about simple everyday actions that can help protect the environment [22].

In partnership with the National Association for the Prevention of Alcoholism and Addiction (l’Association nationale de prévention en alcoologie et addictologie), the National Territorial Health Insurance Fund (la Mutuelle Nationale Territoriale) has developed an application called *Territorial City*, available to local authorities since September **2015**, designed to help with the decision-making process [23]. The scenario proposed to players involves coordinating local personnel and resources in response to an incident linked to the consumption of psychoactive substances. This simulation helps to (1) equip decision makers with the tools they need to prevent and manage risky behavior involving the consumption of psychoactive substances at work and (2) encourage decision makers and their teams to launch preventive actions in real life.

However, as far as we are aware, no serious game had yet been developed to support and help local authorities and regional health care agencies in their efforts to reduce social inequalities in health.

Our contribution to this process, as researchers, involves the operational development of a *research and training* tool fueled by accessible, comprehensible data derived from our own research [9,10]. Our serious game creates a learning environment that encourages players to think about space, territories, and collective learning for effective action.

## Methods

### Description of the Experiment

During the development of this project, an initial meeting was organized to determine the most appropriate manner of proceeding. Several key points were discussed, including (1) how to best adapt this serious game to the various requirements outlined and (2) how to define the role of the various stakeholders in the development process. The second phase involved the creation of the different characters and graphical environments contained in the game, and the game’s structure (decision tree). The third step was to create the associated educational materials in various formats: videos, interviews, animated PowerPoint presentations, and simulations. The last phase was all about finalizing the design, assembly, and settings of the game using Ludiscape.

### Technological Methodology

The system is composed of 2 databases: the first one is used to edit the game, developed in XML, and the second database is for data that are sent over the internet, which is available via a Web-based platform (type: Learning Management System [LMS] Modular Object-Oriented Dynamic Learning Environment [Moodle]) developed in MySQL.

A programming language known as HTML (HTML JavaScript Cascading Style Sheets [CSS]) was used in conjunction with PHP (Hypertext Preprocessor) to develop the activities included in the Web-based system.

### Hardware

This serious game was designed to work on various devices: tablets, PC, and mobile phone. Thanks to the Ludiscape engine, designed for compatibility with all of these devices, no further development was required. This engine is also compatible with all operating systems currently available on the market (Windows, Mac, Linux, iPad and Android tablets, Firefox, and Chrome).

### Game Design Elements

**Figure 1:**
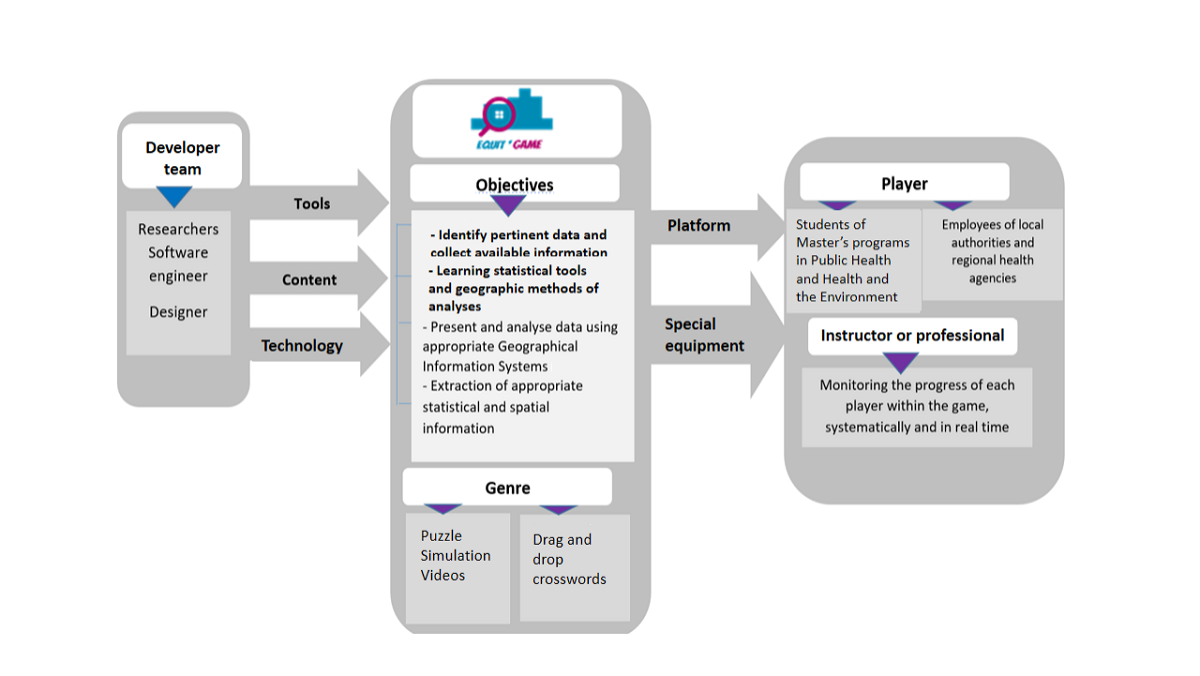
Game design elements.

The creation of our serious game involved different processes, technologies, and specialists. We used the conceptual model developed by Wattanasoontorn et al [16] to illustrate the core components of Equit’Game (Figure 1).

### Development Team

The creation of our serious game brought together a large panel of contributors from different disciplines: scientists, software engineers, graphic designers, simulation analysts, and educators*.* The multidisciplinary expertise of this team enabled us to produce an innovative game featuring interfaces and graphical environments designed to fulfill the objectives of Equit’Game.

### Tools

The development of Equit’Game was based on 2 essential elements: app design software and the game engine. The design software we used is called Ludiscape, created and developed by Damien Renou [24].

**Figure 2:**
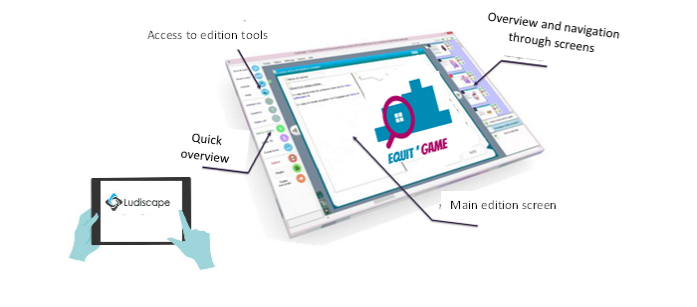
The Ludiscape environment.

Ludiscape is a collection of integrated tools designed to help users create training content, e-learning modules, and serious games. This technology allows users to create a variety of educational content simply by using the Ludiscape resource library. Each object in this library provides quick access to properties and settings, requiring no prior experience in programming (Figure 2). Full integration of JavaScript with the Ludiscape engine and its extensions ensures maximum flexibility and production speed. Plugins are invisibly integrated into the educational production environment in such a way that they are easy to use.

The Ludiscape environment allows users to incorporate educational material in various formats (JPG, WAV, MP3, MP4, and PTT) into a unique project that becomes the game engine. The game engine can then be exported in different formats (HTML, JavaScript, CSS, Shareable Content Object Reference Model [SCORM], EXE, and iOS) toward different platforms (PC, Mac, mobile phone, tablet, phablet, LMS, Moodle, and Dokeos). This unique project contains all of the codes required to control the game and all of the databases that feed into the game. It is this game engine that the graphical user interfaces will open when the user launches the app. On the basis of the decision tree (constructed in one of the first steps of the project), the rules (including the various stages) that announce the success are presented to the learner/player, that is, the levels to be completed to finish the game. When a set of targets have been met, an interface appears to inform the user that the ongoing level is complete and that they can move onto the next level.

### Content

As part of their decision-making process, regional health care agencies and local authorities apply national strategies at the regional and/or local scale to develop targeted local actions. To do this effectively, decision makers require a comprehensive territorial diagnosis based on study, cross-comparison, and precise analysis of the available data.

We, therefore, decided to base our interactive serious game, Equit’Game, on the idea that a comprehensive territorial diagnosis combining health, environmental, and social perspectives would represent an invaluable resource in the decision-making process, helping to boost collective action at the territorial level. To achieve this objective, we need to understand and master the different stages involved in the creation of a territorial diagnosis.

All of the data collected by the Equit’Area project from the City of Paris, involving a fine scale of geographical detail, were used in the development of Equit’Game.

As such, at different levels in the game, we provided players with information and tools developed by scientists and experts. These resources take various forms: statistical indicators, maps, and results of analyses.

To describe the state of health care in Paris, the infant and neonatal mortality rates between 2004 and 2009 have been displayed. Thereafter, the average annual levels of nitrogen dioxide, and particles with a diameter equal to or smaller than 10 µm—as modelled by the agency responsible for monitoring air quality in Greater Paris, AirParif—have been used to visualize the spatial distribution of environmental hazards in Paris (air pollution is only one of the factors used to measure environmental hazards). To finish, the population census data were analyzed to profile the neighborhood socioeconomic deprivation.

### Technology

In creating this game, we opted to use virtual reality technology; this means creating a simulation that gives players the impression that they are present in a real or imaginary environment. Designers and graphical experts created these different environments to represent (1) *virtual spaces*, such as a university campus, a conference center, a classroom, and an office, and (2) *real spaces*, such as conferences by well-known figures and interviews with experts.

### The Equit’Game Tool

Like most serious games in the health care sector, Equit’Game aims to reach several objectives: spreading a message that is informative and educational, while also getting across marketing-persuasive messages intended to induce/influence behavior; in this case, encouraging players to take action and conduct their own territorial diagnosis.

More specifically, the realistic situations employed in our serious game should encourage players, in a fun and playful manner, to (1) appropriate the data of their own territory, (2) apply their methodological knowledge in a practical way, (3) reflect on the most pertinent statistical and/or spatial tools for their situation, and (4), ultimately, to acquire new knowledge and skills in the use of territorial diagnosis tools with a spatial dynamic. These 4 objectives structure our game into 4 levels, as follows:

- Level 1: Dataminer (identifying relevant information to respond to the question);
- Level 2: Analyst (selecting the appropriate method of analysis);
- Level 3: Atlas (mapping the data);
- Level 4: Cluster (extraction of statistical and spatial information).

The serious game was deployed over the course of a week’s training and structured in such a way that each learner should complete 1 level per day. The experience involved a combination of teaching scenarios delivered by a computerized app and can be used with or without an instructor as part of classroom training, distance-learning, or a combination of both.

### Game Formats

The game offers a number of different learning mechanisms, a variety of approaches combined in Equit’Game, and corresponds to the various objectives set for each level. To help players to appropriate the data of their study territory, there are various *hidden areas* that allow players to interact with the territory. There are also crosswords and drag and drop exercises that allow players to apply their methodological knowledge in practical and concrete ways. We also included quiz sections and dialogues involving virtual interaction via text zones to stimulate reflection on the most appropriate statistical and/or spatial tool. Equit’Game also incorporates educational simulations designed to help players acquire new knowledge and skills. Players are allowed to run the simulations several times, using different input data each time.

### Users and Platforms

Our serious game aimed to reach several target groups, including employees of local authorities and regional health agencies. Owing to the diversity and richness of the educational material included in each level, Equit’Game can also be used wholly or partially as part of Master’s programs in public health and/or environmental health.

Users can access Equit’Game via a platform compatible with tablets, PCs, and mobile phones and have to enter their user identification to log in. The platform also allows instructors to monitor the progress of each player within the game, systematically and in real time, with help from the indicators and intermediate scores recorded by the platform.

### Tool Design

#### High-Level System Diagram

Equit’Game is composed of 3 blocks (2 principal components and an interface layer) and includes effective databases in XML and MySQL format.

**Figure 3:**
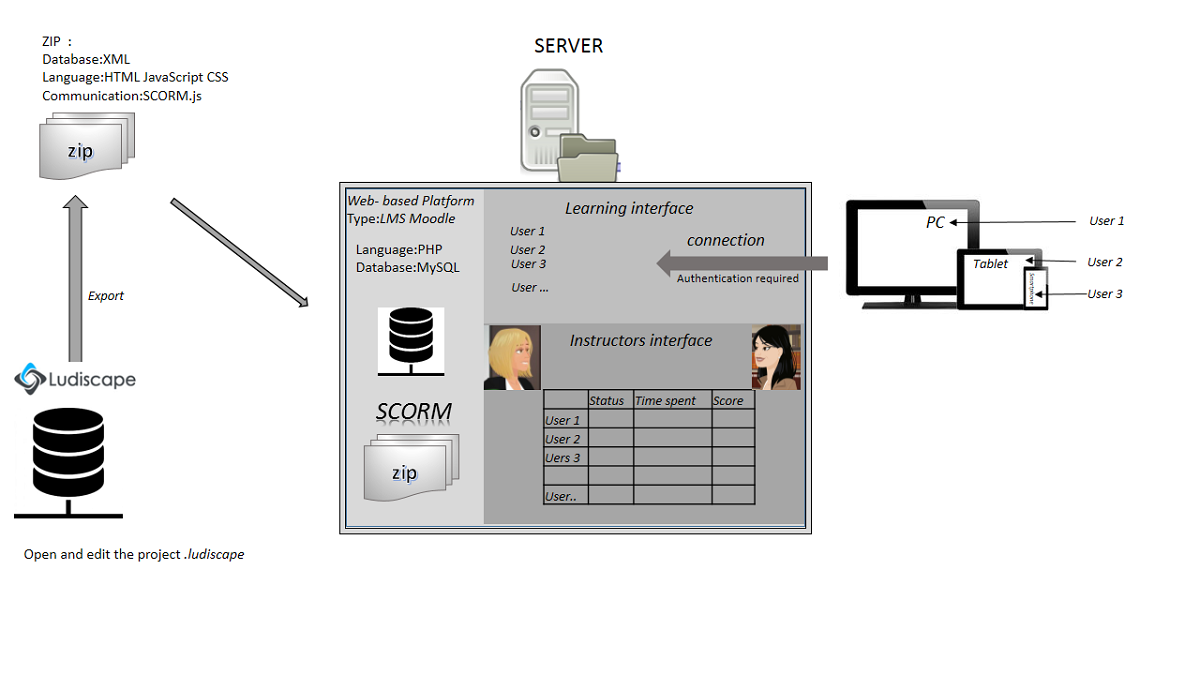
System architecture.

As Figure 3 shows, the structure used allows the Equit’Game administrators to edit the game via the first block, by using an XML database implemented within the Ludiscape software. The game can then be exported as a SCORM.js layer. The SCORM.js interface will be deployed on LMS Moodle or a similar Web-based platform, which operates via the MySQL database and allows players to access Equit’Game.

Therefore, access to Equit’Game, regardless of the device used (PC, tablet, and mobile phone) requires connection to the Web-based platform with personal user ID codes. This final block is where players can save their data to the server via the MySQL database. Instructors can track the progress of players via plugins installed on the Moodle platform, which keep records of different indicators and parameters as users move through the game (eg, time spent on the game and score on each level). Analysis of these indicators, virtually in real time, allows the instructor to follow players through each level of the game and, where necessary, to adapt subsequent training to address any concepts that learners do not seem to have fully understood.

#### Designing Our Serious Game

This serious game is part of the process of translating the results of the Equit’Area research program into training tools. It was designed and developed for the purposes of training users to perform a territorial diagnosis. Equit’Game is thus based on the following key elements: (1) understanding the needs of a territory in the 3 complementary areas of health, environmental exposure, and neighborhood socioeconomic deprivation and (2) learning to handle the data that best characterize the territory, including data collection, transformation, and analysis, and graphical representations and maps.

To fully satisfy the definition of a serious game, *Equit’Game* meets the following criteria:

- Accompaniment in the form of texts and voice messages, guiding the user;
- Use of realistic elements to simulate real cases;
- Clear and easy interfaces;
- No time limits, as users progress at different speeds;
- Congratulations at the end of each level, encouraging players and facilitating the learning process;
- Lack of animation and sound effects to keep players’ attention focused.

**Figure 4:**
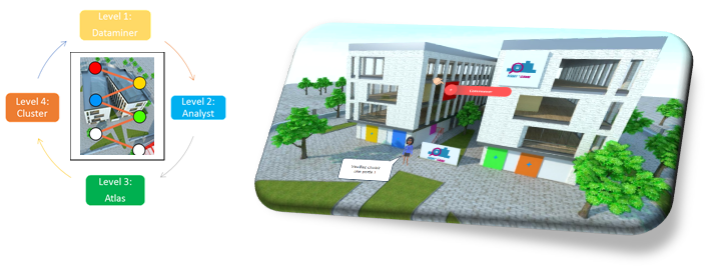
Equit’Game design: (A) structure of 4 levels and (B) fun learning environment: virtual campus.

Equit’Game has also been designed as a sort of *virtual campus*, creating a fun learning environment (Figure 4) in which each door represents a level. The game environment also incorporates an additional space where learners can look back over previous levels to improve their performance, consult the previous learning materials, and save the information they require.

As shown in Figure 4 (screen capture), we have used a color code to identify clearly the 4 levels that make up Equit’Game: level 1 is yellow, 2 is blue, 3 is green, and the fourth and final level is orange. To mimic the structure of a diagnosis focusing on social and/or environmental inequalities relating to health, players can only move onto the next level when they have completed the current one and cannot go backward (Figure 4).

**Figure 5:**
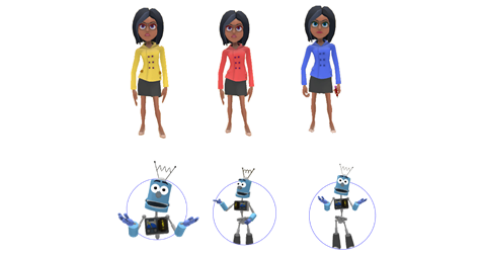
The design of main characters of Equit’Game.

Players encounter different characters throughout the game: the first character, the elegant Marie, welcomes and guides players around the campus at the start of each new level, color-coded accordingly (Figure 5). A second character, Robert the robot, helps players to get to grips with methods and learn how to handle data.

To make Equit’Game more realistic, expert interviews, feedback, and video clips, staged in realistic surroundings, examine the following issues: social health inequalities, environmental inequalities, and health inequalities relating to perinatal health.

#### A Game Constructed Around 4 Levels and 2 Scenarios: Gameplay

Players are presented with 2 scenarios: (1) a diagnosis focusing on socioeconomic factors and health or (2) a diagnosis focusing on environmental factors and health. They must make a choice based on the information provided at the outset of Equit’Game, and the way that the game plays out will change accordingly. For example, if a player decides to focus on environmental inequality, they will have access to the relevant environmental and health data but not the socioeconomic data and vice versa. The structure of each level is described below.

#### Level 1 

In this first level, the goal was to identify pertinent data and to collect the information that is available and is necessary to achieve the stated objective (Multimedia Appendix 1). Players define their objective on the basis of the territory under consideration and the existing literature, justifying their choice.

To this end, the level was broken down into a series of interrelated steps. Upon first connecting to Equit’Game, Marie (a key character in the game) greets the learners and they are taken to a virtual conference where they see several videos. Players must then take part in a type of *board game* exercise, which functions as an evaluation test of knowledge, before leaving the conference. They then set off to *hunt down* the necessary information, taking the train to Paris to gather information on the ground. Back on the campus, they must learn to store these data within a workspace laid out like an office. Various simulations and learning models are available to help learners to construct databases that are understandable and usable for multiple contributors.

Players thus move between 3 game environments (the conference, Paris, and their office; Figure 3), completing 30 challenges (crosswords, drag and drop, and puzzles) along the way to complete this first level. By the time learners reach the end of the first level, they should be capable of choosing the diagnosis they wish to conduct for Paris city and identifying the type of data required to achieve this diagnosis.

#### Level 2

The goal of the second level was to learn how to summarize information using appropriate statistical tools, selected among the available statistical indicators, tables, and graphs (Multimedia Appendix 2). The level was structured as follows: back on campus, Marie (the game’s learning companion; Figure 2) guides the learners toward a blue door, which symbolizes the start of the next level—*Analyst*. Players thus move into a new learning environment, accompanied by blue Marie, and learn about basic statistical methods and the different indicators they can use for territorial diagnosis. Robert is on hand to help learners as they watch 3 tutorials on how to use the statistical software Stata and its main analytical functions. They then get to grips with the software’s various functions and statistical features with the help of crosswords, drag and drop exercises, and puzzles.

To finish off, learners use the various simulation tools to begin processing the data that they themselves collected in level 1, with a view to producing pertinent statistical indicators, constructing graphs, and conducting the necessary statistical analyses (such as a variance analysis or mean comparison test). With Robert’s help, the learners then analyze their results using tools including a quiz and a virtual dialogue session.

Players thus move between 2 game environments (Figure 4): theoretical statistical learning (similar to an actual lecture) and the virtual office (which corresponds more closely to practical work, conducted with or without the help of an instructor).

To complete the level, learners must complete numerous challenges (crosswords, drag and drop, and puzzles). By the end of this level, learners should be able to conduct and interpret statistical analyses on the data they have collected and to present the information clearly.

#### Level 3

The aim of level 3 was to learn how to present data by using appropriate Geographical Information Systems (GISs), and how to interpret the resulting mapping (Multimedia Appendix 3). To achieve this objective, the level was structured as follows: as in the previous level, Marie guides learners across the virtual campus to a green door that opens onto the *Atlas* level. In this new learning environment, green Marie (Figure 5) accompanies players as they learn about GISs, their use, and their importance for territorial diagnosis. Thereafter, Robert (Figure 5) guides players through 5 tutorials focusing on the ArcGIS software and its function in terms of spatial analysis and geomatic and cartographic features. They then familiarize themselves with the use of the various geomatic and spatial mapping functions and features with the help of exercises, including crosswords, drag and drop exercises, and puzzles.

To complete the level, learners must use the various simulation tools at their disposal to map and interpret the data they collected in level 1, producing their own maps to visualize the health spatial distribution and also the environmental or socioeconomic spatial distribution according to their initial choice. With Robert’s help, the learners then analyze their maps using tools, including a drag and drop test, a virtual dialogue session, and a puzzle.

Players thus move between 2 game environments: lessons on the geographical tools (similar to an actual lecture) and the virtual office (which corresponds more closely to practical work, conducted with or without the help of an instructor).

To complete this third level, learners must complete a total of 40 challenges (crosswords, drag and drop exercises, and puzzles). By the end of this level, learners should be able to summarize information using appropriate mapping tools and present essential information clearly.

#### Level 4

The aim of level 4 was to learn how to cross-compare data using appropriate geographical tools and correctly interpret their spatial distribution (Multimedia Appendix 4). To achieve this objective, the level was structured as follows: back on campus, Marie guides the learners toward an orange door, which symbolizes the start of the next level—*Cluster*. In this new learning environment, orange Marie (Figure 5) guides players as they learn the theoretical foundations of clustering techniques, their use, and their importance to establish a territorial diagnosis. Robert (Figure 4) then accompanies them as they watch 4 tutorials on the Satscan software (for scan statistics analysis) and its spatial analysis functions.

To complete the level, learners must use the various simulation tools at their disposal to analyze the data they collected in level 1, identifying the most vulnerable areas of Paris city and disseminating results of the territorial diagnosis. With Robert’s help, the learners then interpret these spatial analyses using tools, including a quiz and a virtual dialogue session.

Players thus move between 2 game environments: a learning environment focused on spatial learning (similar to an actual lecture) and the virtual office (which corresponds more closely to practical work, conducted with or without the help of an instructor).

To complete this fourth level, learners must complete a total of 35 challenges (crosswords, drag and drop exercises, and puzzles). By the end of this level, learners should be able to produce a spatial representation of the data using appropriate geographical tools and present the resulting territorial diagnosis.

### Organization and Management of the Serious Game, Equit’Game

The schematic representation of the structure of Equit’Game, comprise a main block (dispositif.ludiscape) that contains the game’s 4 levels along with external files that contain smaller .ludiscape blocks for each level.

The main block contains all of the levels that make up Equit’Game, including all resources, databases, and videos, and the tests and quiz modules.

The external files contain the tutorials, videos, and simulation modules, incorporating large databases that have been deliberately separated from the main block to reduce the size of the game, facilitating updates, and speeding up the handling of various game scenarios.

## Results

This study was a success because of the cooperation between teachers and researchers from different disciplines, including epidemiology and health geography, specialist of pedagogy and e-learning, and designer of serious games. They participated in the development of all the steps from design to validation of the Equit’Game app.

Our serious game has been presented in several meetings organized in the School of Public Health (École des hautes études en santé publique [EHESP]) and in the Université Sorbonne Paris Cité, who provide financial support for the development of Equit’Game. We also finished the conception of the teaser available on the Web which has been used to communicate the objectives of the game. Several meetings were held with the *Department of Pedagogies*-*EHESP* to validate the development process. In addition, the demo version of the serious game is available to all on the Web [25].

In the first step, we planned to test our app interface, designed especially for adults (professional and postgraduate students) among a panel of local professionals who work directly with interested students and professionals. These are some of the most relevant points:

- Font size and colors used.
- Voice accompaniment in texts and messages, guiding the user.
- Use of realistic elements to simulate a real case.
- Clear and easy interfaces.
- No time limits.
- The change between successive game levels.
- Conclusions after each game level to encourage users and facilitate the learning process by repeating concepts.
- Animations and sound effects to keep the attention of the user.

In the second step, we planned to use our application, Equit’Game, with postgraduate students of EHESP in the next academic year. This educational development will be part of different modules. It will consolidate teaching modules of spatial analysis, information systems, biostatistics, and risk assessment related to the urban environment toward students enrolled in the Master of Public Health and Environmental Risks (about 20-25 students/year). It will also be a part of the teaching in the Master’s degree in Engineering and Risk Management in Environmental and Occupational Health, particularly within the *Spatial approach as a tool for territorial diagnosis* module coordinated by the bearer of this project (about 10-15 students/year).

The variables analyzed concern both the application interface and the running of the game. The point studies were time, number of attempts, and errors.

All the sessions, both for the design and system validation, will be carried out during the class with the presence of a teacher.

At the end of the game session, we will perform a satisfaction survey, including several items:

- Did you like Equit’Game and its different interfaces?
- Whether they would play again (Will you play again?);
- Did you find the game complicated?
- Did you have fun? (whether the users had fun playing);
- Did you know/have you ever played with serious games before?

The teacher will detail all the questions orally in a clear and simple way to facilitate the level of understanding of all users. Then, users can answer all questions on the Web.

Some items will be evaluated with binary answers (Yes or No), while others assess the level of difficulty using a 10-point scale.

In addition, from our platform, which allows us to monitor the progress of each player within the game, an objective assessment based on parameters implemented in the system can be made, including time spent in performing the different activities and number of attempts or level played.

In the long term, this original development will be adapted to propose the game as an e-learning program.

## Discussion

Equit’Game was developed to help learners with the techniques of territorial diagnosis, with the aim of creating an *innovative tool for public health* capable of conveying educational messages and providing a structure for training. Equit’Game provides a fun learning environment in which players learn how to approach a territory and conduct spatial analyses by using a combination of methods, tools, and data, ensuring that information is not misinterpreted. This educational tool can be adapted to other territories, and the approach adopted in this project is transposable in other public health issues.

## Appendix

Multimedia Appendix 1 Equit’Game level 1.

Multimedia Appendix 2 Equit’Game level 2.

Multimedia Appendix 3 Equit’Game level 3.

Multimedia Appendix 4 Equit’Game level 4.

## Abbreviations

ADEME: Agence de l'Environnement et de la Maîtrise de l'Énergie
CSS: Cascading Style Sheet
EHESP: École des hautes études en santé publique
GIS: geographical information system
LMS: learning management system
Moodle: modular object-oriented dynamic learning environment
SCORM: shareable content object reference model
