# Supplementary figures and images for: Development of Serious Games (Equit’Game) to Address Health and Environmental Inequalities: Protocol for an App-Delivered Program to Perform a Territorial Diagnosis
Source: JMIR Res Protoc. 2020 Jan 7;9(1):e11786. doi: 10.2196/11786 (PMC6996730; doi:10.2196/11786)

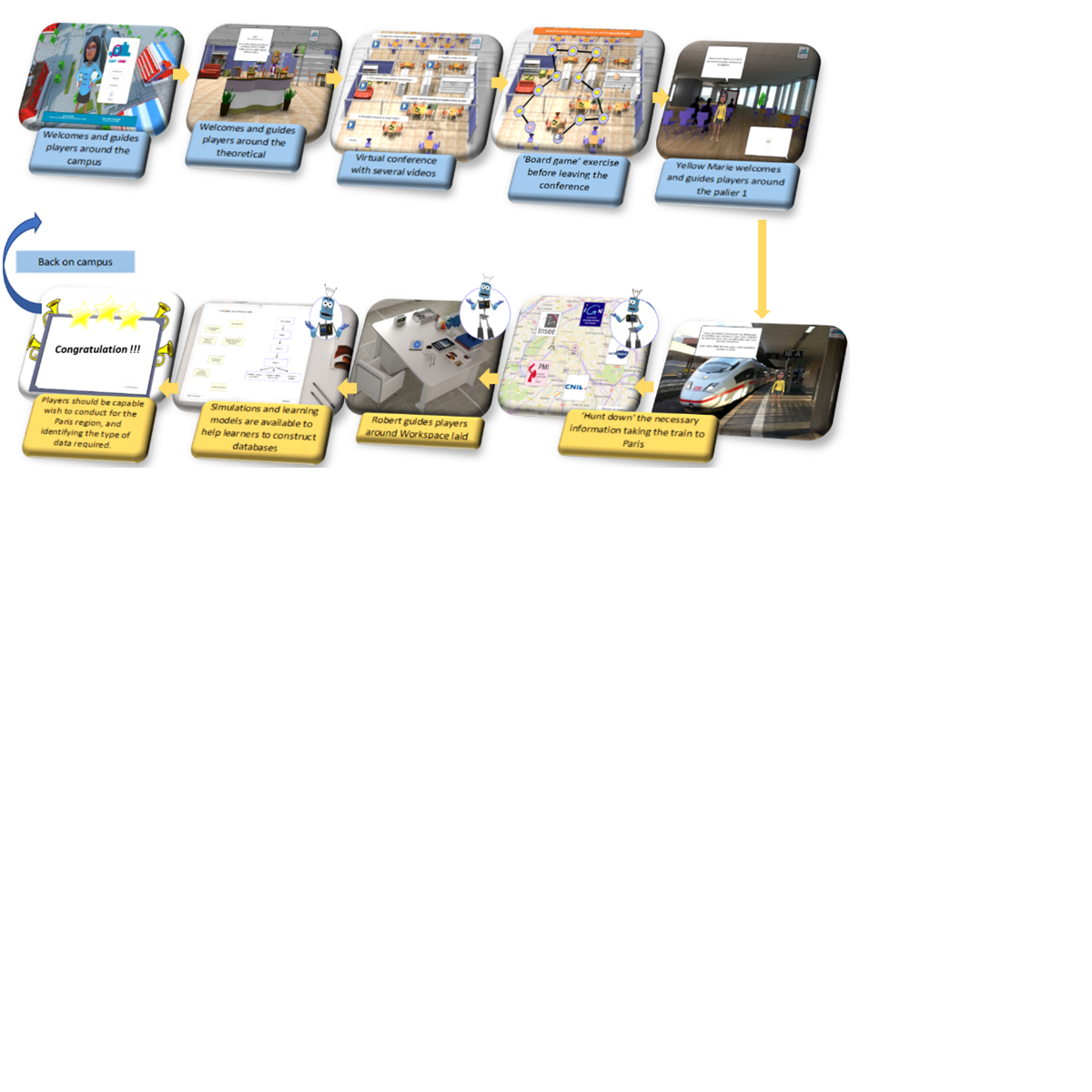

Supplement: Multimedia Appendix 1 [file resprot_v9i1e11786_app1.png]

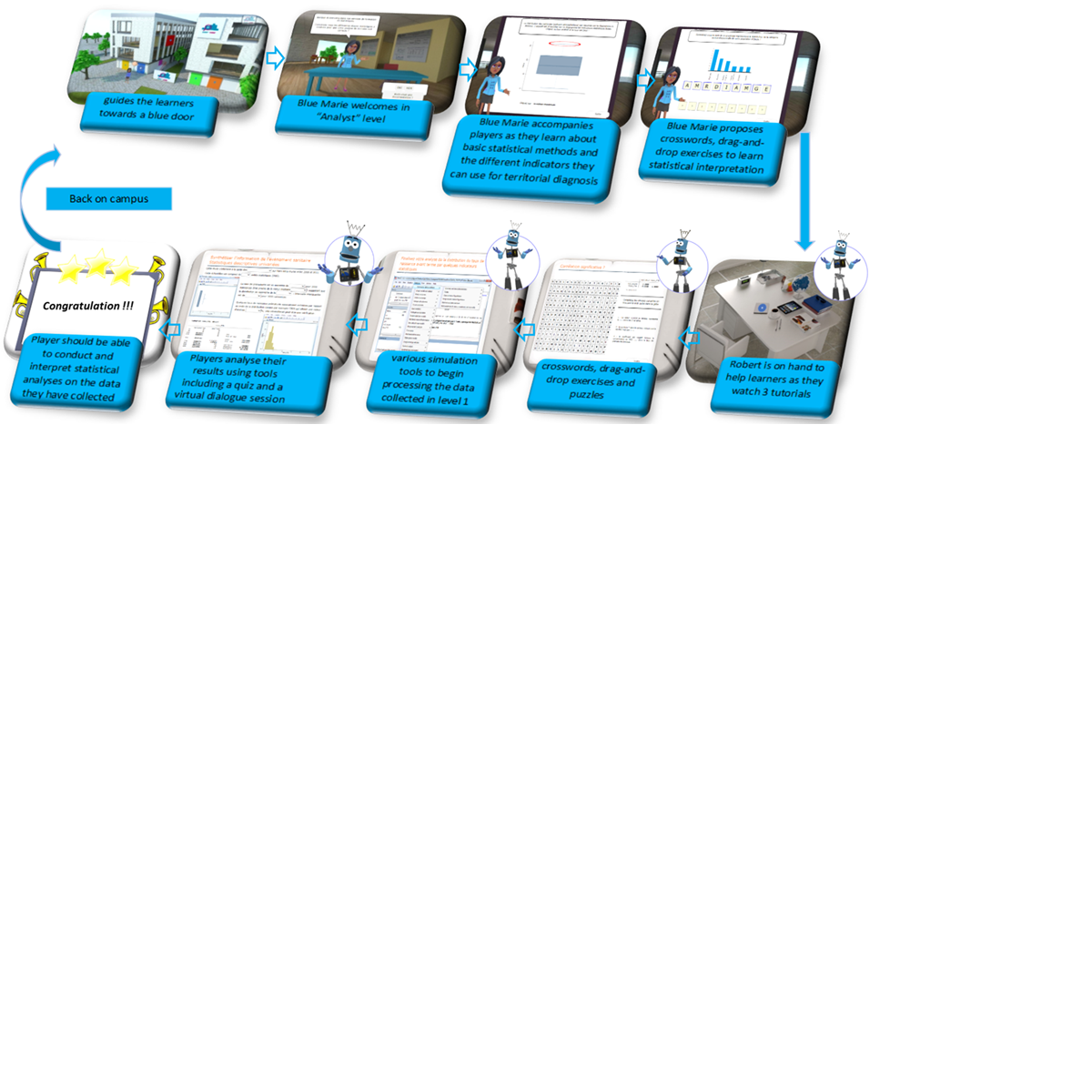

Supplement: Multimedia Appendix 2 [file resprot_v9i1e11786_app2.png]

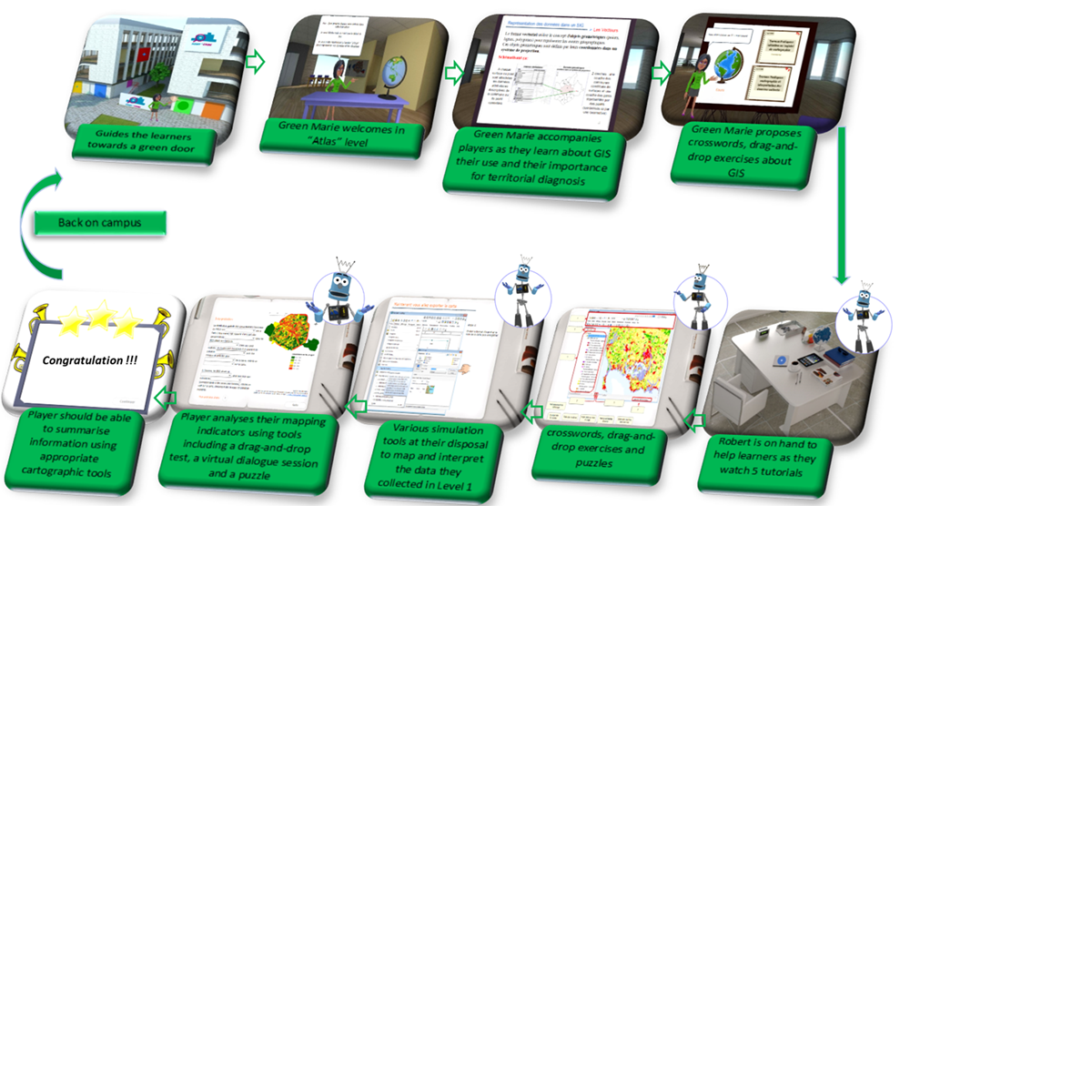

Supplement: Multimedia Appendix 3 [file resprot_v9i1e11786_app3.png]

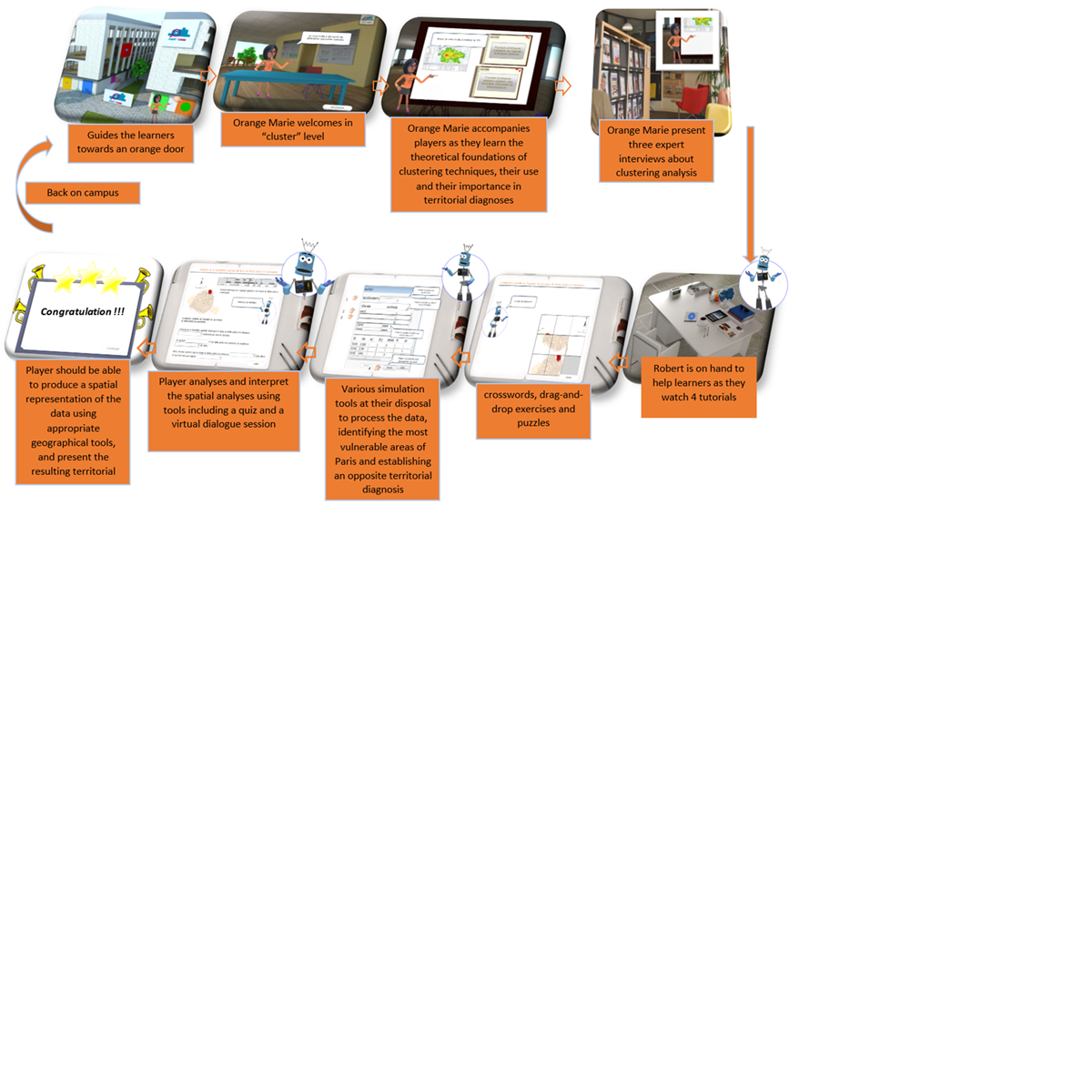

Supplement: Multimedia Appendix 4 [file resprot_v9i1e11786_app4.png]
